# Inward Displacement: A Novel Method of Regional Left Ventricular Functional Assessment for Left Ventriculoplasty Interventions in Heart Failure with Reduced Ejection Fraction (HFrEF)

**DOI:** 10.3390/jcm12051997

**Published:** 2023-03-02

**Authors:** Romy R. M. J. J. Hegeman, Sean McManus, Jan-Peter van Kuijk, Serge C. Harb, Martin J. Swaans, Patrick Klein, Rishi Puri

**Affiliations:** 1 Department of Cardiothoracic Surgery, Sint Antonius Hospital Nieuwegein, 3435 CM Nieuwegein, The Netherlands; 2 Bioventrix Inc., San Ramon, CA 94583, USA; 3 Department of Cardiology, Sint Antonius Hospital Nieuwegein, 3435 CM Nieuwegein, The Netherlands; 4 Department of Cardiovascular Medicine, Heart, Vascular & Thoracic Institute, Cleveland Clinic, Cleveland, OH 44195, USA

**Keywords:** hybrid left ventricular reconstruction, ischemic heart failure, ischemic cardiomyopathy, left ventricular remodeling, minimally invasive cardiac surgery, inward displacement, speckle tracking echocardiography, strain

## Abstract

Background: Hybrid minimally invasive left ventricular reconstruction is used to treat patients with ischemic heart failure with reduced ejection fraction (HFrEF) and antero-apical scar. Pre- and post-procedural regional functional left ventricular assessment with current imaging techniques remains limited. We evaluated ‘inward displacement’ as a novel technique of assessing regional left ventricular function in an ischemic HFrEF population who underwent left ventricular reconstruction with the Revivent System. Methods: Inward displacement adopts three standard long-axis views obtained during cardiac MRI or CT and assesses the degree of inward endocardial wall motion towards the true left ventricular center of contraction. For each of the standard 17 left ventricular segments, regional inward displacement is measured in mm and expressed as a percentage of the maximal theoretical distance each segment can contract towards the centerline. The left ventricle was divided into three regions, obtaining the arithmetic average of inward displacement or speckle tracking echocardiographic strain at the left ventricular base (segments 1–6), mid-cavity (segments 7–12) and apex (segments 13–17). Inward displacement was measured using computed tomography or cardiac magnetic resonance imaging and compared pre- and post-procedurally in ischemic HFrEF patients who underwent left ventricular reconstruction with the Revivent System (*n* = 36). In a subset of patients who underwent baseline speckle tracking echocardiography, pre-procedural inward displacement was compared with left ventricular regional echocardiographic strain (*n* = 15). Results: Inward displacement of basal and mid-cavity left ventricular segments increased by 27% (*p* < 0.001) and 37% (*p* < 0.001), respectively, following left ventricular reconstruction. A significant overall decrease in both the left ventricular end systolic volume index and end diastolic volume index of 31% (*p* < 0.001) and 26% (*p* < 0.001), respectively, was detected, along with a 20% increase in left ventricular ejection fraction (*p* = 0.005). A significant correlation between inward displacement and speckle tracking echocardiographic strain was noted within the basal (R = −0.77, *p* < 0.001) and mid-cavity left ventricular segments (R = −0.65, *p* = 0.004), respectively. Inward displacement resulted in relatively larger measurement values compared to speckle tracking echocardiography, with a mean difference of absolute values of −3.33 and −7.41 for the left ventricular base and mid-cavity, respectively. Conclusions: Obviating the limitations of echocardiography, inward displacement was found to highly correlate with speckle tracking echocardiographic strain to evaluate regional segmental left ventricular function. Significant improvements in basal and mid-cavity left ventricular contractility were demonstrated in ischemic HFrEF patients following left ventricular reconstruction of large antero-apical scars, consistent with the concept of reverse left ventricular remodeling at a distance. Inward displacement holds significant promise in the HFrEF population being evaluated pre- and post-left ventriculoplasty procedures.

## 1. Introduction

Hybrid minimally invasive and fully percutaneous transcatheter therapies are rapidly evolving to target the ever increasing and comorbid heart failure with reduced ejection fraction (HFrEF) population, many of whom do not meet the criteria for advanced HF therapies or transplantation, yet continue to suffer from poor quality of life (QOL) and repeated HF hospitalizations [1]. These evolving therapies are aimed at physical reverse left ventricular (LV) remodeling and have specific targets along the LV based on HFrEF etiology and the device’s mechanism of action. The Revivent TC^TM^ therapy (BioVentrix Inc., San Ramon, CA, USA) has evolved to effectively target ischemic HFrEF patients with antero-apical scar folding and volume reduction via pledgeted anchors, while the AccuCinch^®^ Ventricular Restoration System (Ancora Heart Inc., Santa Clara, CA, USA) typically targets the LV base in non-ischemic HFrEF via endocardial anchoring of a non-circumferential ring that is cinched to reduce the septo-lateral LV diameter [2,3]. Pivotal trials of both the Revivent and AccuCinch devices are ongoing to evaluate the incremental clinical benefit of these therapies on top of HFrEF guideline-directed medical therapy (GDMT). Various other therapies (i.e., V-Sling (Cardiac Success Ltd., Yorkneam, Israel), transcatheter transmyocardial Algisyl alginatehydrogel therapy (LoneStar Heart Inc., Laguna Hills, CA, USA), Heart Damper (Eucardia, Milan, Italy)) remain in early clinical development [4]. The key to patient selection and post-procedural assessment with these evolving ventriculoplasty therapies is the ability to reliably identify and accurately assess regional LV structure and function at multiple time points.

Echocardiography remains the current means of diagnosing/identifying HFrEF individuals, yet lacks the ability to reliably reproduce the quality of imaging required to truly evaluate regional LV structure and function pre- and post-LV ventriculoplasty procedures with the precision required to better understand the mechanism and efficacy of such therapies. Volume and EF measurements of the LV in standard two-dimensional (2D) echocardiography are associated with a high uncertainty due to interobserver variability of the manual measurement, but also due to ultrasound acquisition errors such as apical foreshortening [5,6]. In expert centers with state-of-the-art imaging with advanced computational image processing algorithms, three-dimensional speckle tracking echocardiography (3D STE) has been used to unravel the concept of reverse LV remodeling at a distance in patients treated with surgical LV reconstruction for their ischemic cardiomyopathy with large apical scars [7]. However, novel imaging methods that are more reproducible could complement echocardiography in the framework of multimodality imaging in HFrEF patients.

Computed tomography (CT) and cardiac magnetic resonance (CMR) imaging have inherent advantages in their ability to reliably identify and project the 17 LV segments sans foreshortening at any time point and without any of the other TTE limitations [5]. We describe inward displacement (InD), a novel means of assessing regional LV function using pre- and post-procedural CT or CMR imaging in ischemic HFrEF patients with large anteroseptal and/or apical scars who underwent left ventricular reconstruction (LVR) with the Revivent TC^TM^ system. We describe the InD methodology, its application to HFrEF patients pre- and post-left ventriculoplasty, its correlation with STE and its future potential role in managing HFrEF patients being assessed for device-based therapies.

## 2. Materials and Methods

### 2.1. Inward Displacement

**Figure 1:**
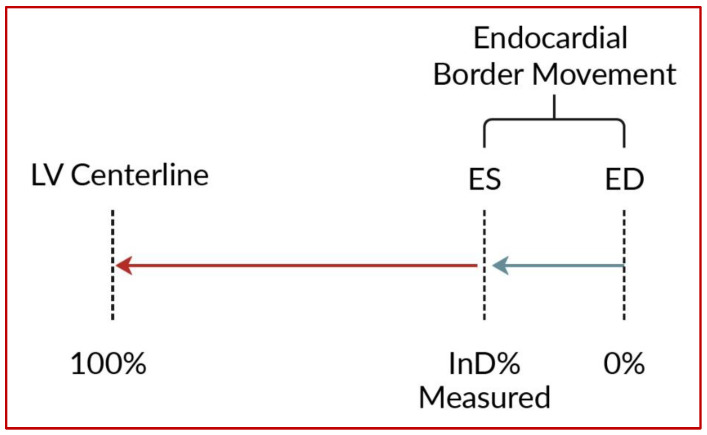
Regional InD is measured in mm and expressed as a percentage. Within the range of InD, the absence of contraction is expressed as 0%, whereas negative percentages imply dyskinesia of the LV wall. InD of 100% corresponds to a theoretical limit at which the LV shrinks to zero volume upon its centerline.

InD represents the effective result of total LV contraction, comprising longitudinal and radial motion towards the LV center. The true centerline of the LV is located midway between the end-diastolic and end-systolic contours of the LV [8,9]. InD is calculated from three standard long-axis views obtained during CMR or CT and assesses the degree of inward endocardial wall motion from end diastole until end systole towards this true LV center of contraction (Figure 1).

**Figure 2:**
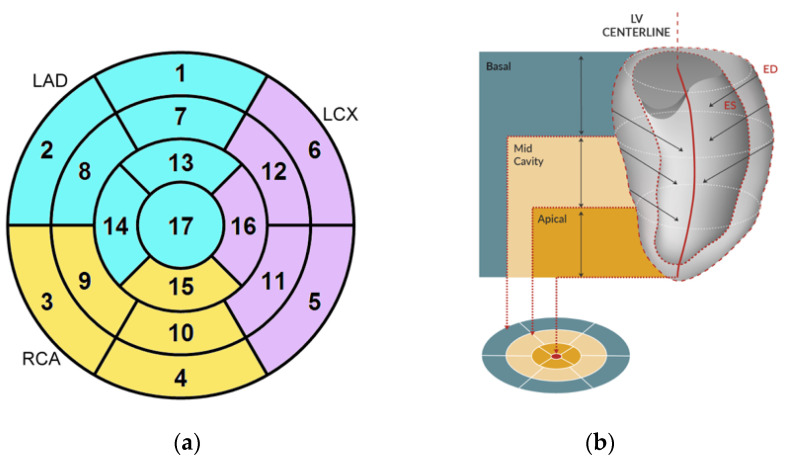
(**a**) Standard AHA Guidelines bullseye plot used for LV assessment, showing the 17 segments. (**b**) Segments are categorized into three LV regions (basal, mid cavity and apical) for regional InD. InD was quantified by measuring the distance travelled by the endocardial tracked points towards the center of the left ventricle during systole (i.e., from end diastole [ED] to end systole [ES]).

For each of the standard 17 LV segments of the standard AHA Guideline bullseye plot [10] (Figure 2a), InD was measured in mm and expressed as a percentage. The absence of segmental LV contraction was expressed as 0% (i.e., akinesia), whereas negative percentages imply dyskinesia of the LV wall. InD of 100% corresponds to a theoretical limit at which the LV shrinks to zero volume. The LV was divided into 3 regions, obtaining the arithmetic average of InD at the LV base (segments 1–6), mid-cavity (segments 7–12) and apex (segments 13–17) (Figure 2b).

### 2.2. CMR and CT Analyses

**Figure 3:**
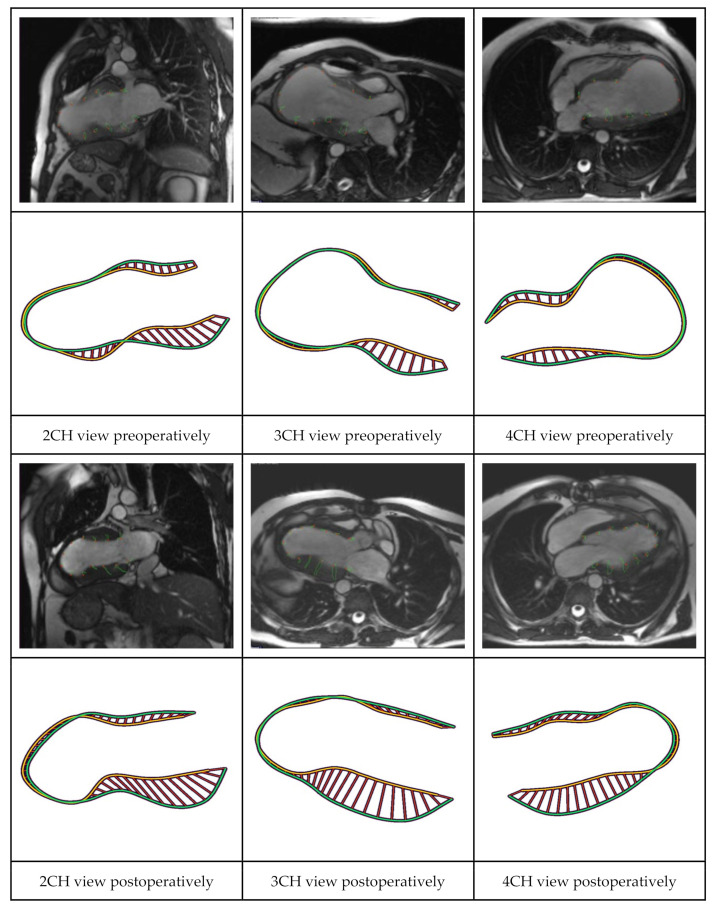
Case example (1) showing the long-axis views on CMR that are used for InD calculation. From left to right: 2CH, 3CH and 4CH views on CMR. The overlapping three long-axis views are shown both preoperatively (row 1) and postoperatively (row 3) (i.e., at the same moment of the cardiac contraction cycle), with corresponding InD (row 2 and 4, respectively). The images identify the increase in InD (displayed by the striped myocardial region) of each of the LV regions following hybrid LVR. Abbreviations: CMR, cardiac magnetic resonance; CH, chamber; InD, inward displacement; LV, left ventricular; LVR, left ventricular reconstruction.

For each subject, the three LAX image series (2CH, 3CH and 4CH) from CT or CMR scans were transferred to an image analysis workstation (MedisSuite MR 2022, Medis Medical Imaging Systems BV, Leiden, The Netherlands). Automated endocardial border LV contour detection was conducted for each of the LAX series for the end-diastolic (ED) and end-systolic (ES) frames. If needed, manual corrections were performed on the automatically detected endocardial contours. Next, the InD Feature Tracking computation (Figure 3) was performed using the QStrain application (Medis Medical Imaging BV, Leiden, The Netherlands) [11].

### 2.3. Speckle Tracking Echocardiography

The 2D STE is a gray-scale based angle-independent method for myocardial strain measurement that is used to estimate deformation measures and quantitatively characterize LV function [12]. A gray-scale image on echocardiography is composed of multiple bright speckles that come from the scatter of the ultrasound beam by the tissue. The speckles are identified and tracked frame-by-frame. From this data, the STE software automatically resolves the magnitude of myocardial deformation in different directions and generates strain and strain rate curves. There are three principal types of strain: longitudinal, circumferential and radial or transmural strain. The longitudinal strain is measured from the apical long-axis images, whereas the short-axis images are used for measuring radial and circumferential strain and rotation [13].

In this study, speckle tracking was performed with the AutoStrain LV software (TOMTEC Imaging Systems GmbH, Unterschleißheim, Germany). The data were analyzed in terms of end-systolic longitudinal strain (ESLS), with longitudinal strain being measured at the endocardial border. Segmental strain values were then displayed on the bullseye plot.

### 2.4. Study Population

InD was analyzed across two groups of HFrEF patients who underwent LVR with the first or second generation Revivent System. All studied patients were operated on after maintaining optimized GDMT for at least 90 days. GDMT included a combination of beta-blockers, angiotensin-converting enzyme (ACE) inhibitors, angiotensin receptor blockers (ARB), angiotensin receptor neprilysin inhibitors (ARNI) and/or mineralocorticoid receptor antagonists (MRA). The first group comprised patients who were analyzed for CE-mark certification [14]. Of 86 patients who underwent LVR in this group, only patients for whom both pre- and postoperative CT- or CMR-scans were available and image quality was sufficient were included for analysis. A second group of hybrid LVR patients who were operated on at the St. Antonius Hospital Nieuwegein, the Netherlands, and for whom pre- and postoperative CT- or CMR-scans were available with sufficient image quality, were analyzed. This group also underwent pre-procedural STE.

### 2.5. Statistical Analysis

Continuous data are presented as mean ± standard deviation (SD) or as median (interquartile range [IQR]). Categorical outcomes were expressed as frequencies and percentages. Preoperative and postoperative continuous, normally distributed data of the same patients were compared using the paired Student’s *t*-test. Non-normally distributed data were compared using the Wilcoxon signed rank test for paired samples. Categorical outcomes were analyzed using the Wilcoxon signed rank test. Two major analyses were made. With the first analysis, InD was analyzed and compared pre- and postoperatively for both study cohorts. With the second analysis, pre-procedural InD was compared with pre-procedural STE and correlations were assessed. Scatterplots were drawn to identify a linear relationship between STE and InD measurements at baseline. Additional Pearson’s correlation analyses were performed to detect the strength of correlation. Bland Altman plots were constructed to investigate any possible relationship of the discrepancies between the absolute values measured with InD and STE.

## 3. Results

### 3.1. Patient Selection

**Figure 4:**
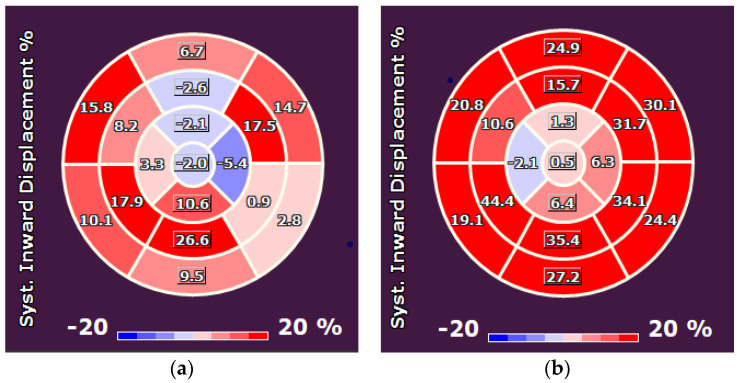
Comparison of preoperative (**a**) and postoperative (**b**) case example. InD is quantified by measuring the distance travelled by the endocardial tracked points towards the center of the left ventricle during systole (i.e., from end diastole to end systole). InD of each segment is measured, and results are plotted on a standard 17-segment model. The displacement is expressed in %. Darker red segments indicate good contractility of the LV wall; lighter red indicate regions of hypokinesia; whereas blue segments represent regions of dyskinesia. InD percentages towards 0% indicate progressive hypokinesia; InD percentages around 0% show akinesia; InD percentages below 0% indicate dyskinesia.

**Figure 5:**
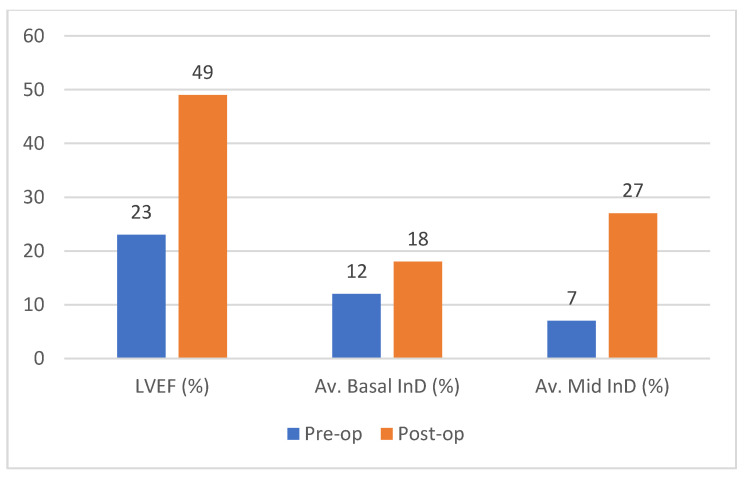
Case example of CMR-derived measurements: overview of improvement of LVEF (%), average basal InD (%) and average mid-cavity InD (%) of the LV. Abbreviations: CMR, cardiac magnetic resonance imaging; InD, inward displacement; LVEF, left ventricular ejection fraction.

Out of a total group of 86 patients who underwent LVR with the Revivent System in the CE-mark cohort, 21 patients were included for InD analysis pre- and post-LVR. Out of 30 patients who were operated on at the St. Antonius Hospital with the Revivent System, 15 patients were included for InD analysis pre- and post-LVR. InD was thus derived from CT- and/or CMR-images and analyzed both pre- and postoperatively at the LV base (segments 1–6) and mid-region of the LV (segments 7–12) in 36 patients (Figure 4 and Figure 5). The included patients (29 males, 7 females; mean age 67 ± 15 years) had a mean left ventricular ejection fraction (LVEF) of 31 ± 9% and left ventricular end systolic volume index (LVESVi) of 67 ± 28 at baseline.

### 3.2. Analysis 1: InD Pre- and Post LVR

**Figure 6:**
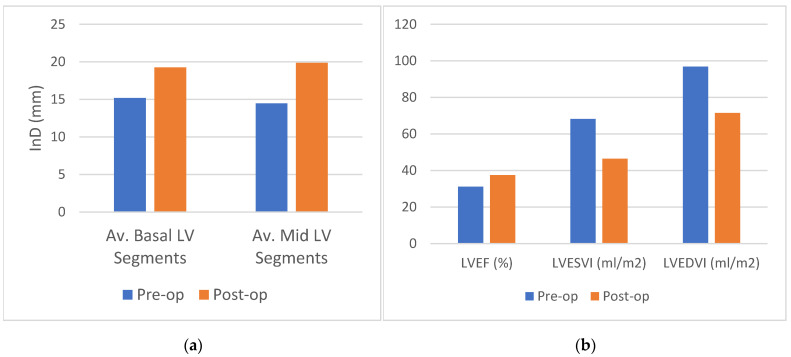
(**a**) Comparison of average pre- and postoperative inward displacement in mm for 36 patients. In the basal left ventricular (LV) segments, a 27% increase in function was found (*p* < 0.001). In the mid LV segments, a 37% increase in function was found (*p* < 0.001). (**b**) Comparison of volumetric pre- and postoperative echocardiographic data measured after a median period of 11 months (IQR 7–27). A significant decrease in LVESVi and LVEDVi was measured of 31% (*p* < 0.001) and 26% (*p* < 0.001), respectively, in addition to a 20% increase in LVEF (*p* = 0.005). Abbreviations: LV, left ventricular; LVEDVi, left ventricular end diastolic index; LVESVi, left ventricular end systolic index; mm, millimeter; post-op, postoperatively; pre-op, preoperatively.

Preoperatively, InD was derived from CT in 9 cases (25%) and from MRI in 27 cases (75%). Postoperatively, InD was derived from CT in 18 cases (50%) and from MRI in 18 cases (50%). Follow-up CT or MRI were performed after a median period of 11 months (IQR 7–27). Comparing InD pre-LV reconstruction with corresponding post-LV reconstruction imaging in 36 patients, mean InD increased significantly in the basal LV region from 15.2 ± 5.8 to 19.3 ± 6.3 (27% increase, *p* < 0.001) (Figure 6a). InD in the mid-region of the LV increased from 14.5 ± 5.4 to 19.9 ± 7.8 (37% increase, *p* < 0.001). LVESVi decreased from 68.2 ± 27.6 to 46.4 ± 24.3 (31% decrease, *p* < 0.001); left ventricular end diastolic volume index (LVEDVi) decreased from 96.8 ± 30.7 to 71.5 ± 26.4 (26% decrease, *p* < 0.001) (Figure 6b). In this cohort, the LVEF increased from 31.1 ± 9.1 to 37.4 ± 13.8% (20% increase, *p* = 0.005).

### 3.3. Analysis 2: Correlation of Baseline InD with STE

**Figure 7:**
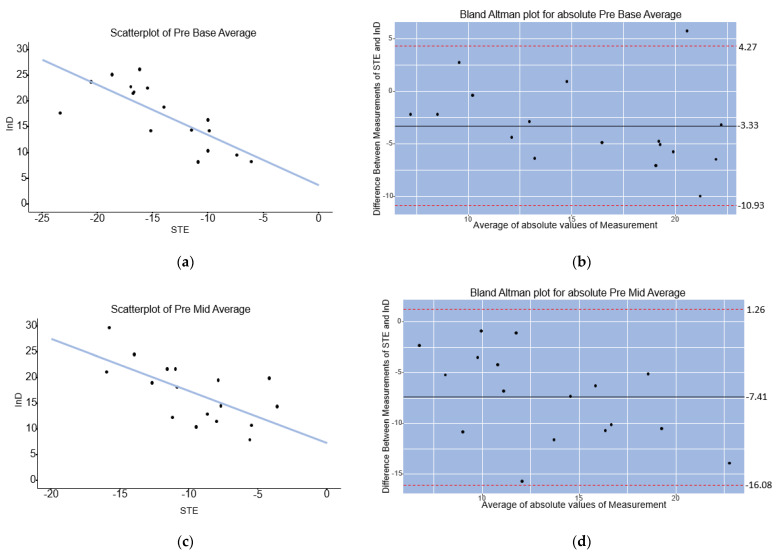
(**a**) Scatterplot showing a significant linear relationship (R = −0.77, *p* < 0.001) between InD and STE in the LV base (segments 1–6). (**b**) Bland Altman plot showing the mean difference of absolute values of InD and STE for the LV base. (**c**) Scatterplot showing a significant linear relationship (R = −0.65, *p* = 0.004) between InD and STE in the mid-cavity (segments 7–12) of the LV. (**d**). Bland Altman plot showing the mean difference of absolute values of InD and STE for the LV mid-cavity.

In 17 patients, both STE and InD measurements were performed at baseline prior to hybrid LVR with the Revivent System. A linear relationship was found between InD and STE in the scatterplots for the LV base and mid-cavity (Figure 7a,c). Pearson’s correlation analyses indicated that there was a strong and significant correlation between InD and STE in the LV base and mid-cavity, respectively, R = −0.77, *p* < 0.001 and R = −0.65, *p* = 0.004. Bland Altman analysis showed that InD measurements result in larger absolute values compared to STE measurements, with a mean difference of absolute values of InD and STE of −3.33 for the LV base, with 95% limits of agreement of −10.93 to 4.27 (Figure 7b). For the LV mid-cavity, Bland Altman analysis showed a mean difference of absolute values of InD and STE measurements of −7.41, with 95% limits of agreement of −16.08 to 1.26 (Figure 7d).

## 4. Discussion

The present study describes the utility of InD, a novel CT/CMR-based methodology of accurate segmental LV assessment. Using a LVR population with ischemic HFrEF with large anterolateral and/or apical scars, we demonstrate the accuracy of the InD technique to elucidate how the LV adapts regionally to a ventriculoplasty procedure delivered at the LV apex. The concept of reverse LV remodeling at a distance was clearly demonstrated, whereby each of the LVR recipients showed a significant increase in the InD of the basal and mid-cavity segments following LV scar plication, contributing to global LV volume reductions. These changes in segmental InD correlated with overall LV volume reductions. InD correlated with STE measures of longitudinal shortening on TTE. These nascent observations demonstrate InD to hold significant promise in the evaluation and planning of ventriculoplasty therapies for HFrEF patients, as well as for post-procedural assessment of loco-regional treatment efficacy, overcoming the shortfalls and limitations of surface echocardiography.

Although LVR with both the first and second generation Revivent System, along with other emerging left ventriculoplasty procedures are shown to be effective [14,15,16], accurate and reproducible evaluation of regional/segmental LV functional improvement post-left ventriculoplasty with current imaging remains challenging. GLS was incorporated as an alternative to LVEF to determine the overall systolic LV function [17], yet does not differentiate between the basal, mid and apical regions of the LV cavity. Since the apex is plicated during hybrid LVR with the Revivent System and the LV base is left untouched by this procedure, contractility of the residual basal and mid-ventricular cavity is of interest when identifying procedural candidates and quantifying regional functional improvement post-procedurally. In order to differentiate between the contractility of all individual regions of the LV, imaging methods are required that enable segment-specific tracking of the motion of the endocardial wall or border. Among these methods, speckle-tracking echocardiography is an accepted means of assessing LV function and can differentiate between the specific LV segments by tracing the inward motion of the endocardial wall. Nevertheless, CMR and CT are widely regarded as offering more accurate imaging than TTE, without the known shortfalls of TTE, which include its relatively poor reproducibility and imaging foreshortening, especially in patients with limited or poor acoustic windows, let alone sonographer-to-sonographer variability. Hence, CMR and CT derived InD was recently developed to measure the regional displacement of the endocardial border towards the LV centerline with respect to the standard three long-axis views. This overcomes the inaccuracies of currently available CT/CMR strain packages.

Thus far, only a single case report has been published describing CT-derived InD as a tool for quantification of segmental LV wall motion abnormalities [11]. The current study describes the utility of InD, a novel CT/CMR-based methodology enabling accurate segmental LV assessment pre- and post-left ventriculoplasty. Using a LVR population with ischemic HFrEF consequent to the development of large anteroseptal and/or apical scars, we demonstrate the accuracy of the InD technique to elucidate how the LV adapts regionally to a ventriculoplasty procedure delivered uniquely at the LV apex. We evaluated InD across two patient cohorts, describing 36 patients pre- and post-LVR. Retrospective analysis of each of the LVR recipients revealed a significant improvement in InD of the basal and mid-cavity segments following the apical folding of the LV scar, contributing to global LV volume reduction and LVEF improvement, indicative of reverse LV remodeling at a distance. These findings are in line with the observations of Castelvecchio and colleagues who demonstrated similar findings using advanced computational finite element image processing from 3D STE [7]. In our combined cohorts, a 27% increase (*p* < 0.001) was found in basal LV contractility in addition to a 37% increase in contractility of the mid-cavity (*p* < 0.001), with an overall 31% and 26% reduction in LVESVi and LVEDVi, respectively, that coincided with a 20% LVEF improvement. Furthermore, InD correlated significantly with STE measures of longitudinal shortening on TTE.

Eventually, InD might supersede STE as the gold standard for assessing regional/segmental LV function, since crisp endocardial border detection and precise means of pre- and post-procedural image comparisons using an automated image processing system minimize the shortcomings of TTE at two (or more) time points. The present Bland Altman analyses suggest InD values tend to be greater than corresponding values measured with STE, which is not surprising given the lack of foreshortening with CT and CMR. Further validation in identifying the InD reference ranges for each of the 17 LV segments across larger numbers of control subjects is currently underway to better place the current observations into clinical context. Overall, these nascent observations demonstrate InD to hold significant promise in the evaluation and planning of left ventriculoplasty therapies for HFrEF patients, as well as for post-procedural assessment of loco-regional treatment efficacy, overcoming the shortfalls and limitations of surface echocardiography.

### Limitations

The present analysis is an observational retrospective study. As the availability of CT and CMR images differed per patient, two different imaging modalities were used for InD analyses. Moreover, as pre- and postoperative CT or CMR images were not made in all patients, InD data were not available for all patients, finally resulting in the inclusion of 36 patients in this study. Furthermore, STE images were not available for the CE-mark cohort, preventing a larger analysis of correlations between baseline STE and InD in this study. Although the number of patients in both cohorts remains relatively small, the present analysis represents the single and largest patient population to date in which InD has been measured, comprising the only currently available data of its application in a HFrEF population that underwent a left ventriculoplasty intervention, enabling the unique opportunity to assess regional LV segmental remodeling at a distance. As functional symptomatic status (expressed with New York Heart Association class, Six Minute Walk Test and heart failure questionnaires) was not available, it was not possible to translate improvement in InD to an improvement in clinical functional outcome. However, the InD findings post-left ventriculoplasty do correlate well with the significant LV volume reductions observed in the patient population, which serves as an important surrogate endpoint of clinical efficacy in the HFrEF population independent of LVEF [18]. As InD data in a healthy control group were not available, InD in this HFrEF cohort could not be compared with InD data in the healthy LV. Moreover, the reference ranges for InD across all LV segments require further validation in a large cohort of control subjects (age and sex-matched) who underwent CT or CMR imaging. Such an analysis is presently underway.

## 5. Conclusions

InD, measured using CT or CMR, successfully demonstrated the concept of reverse LV remodeling at a distance when an ischemic HFrEF population with large anteroseptal and/or apical scars underwent a hybrid LVR procedure. The ability of InD to reliably and reproducibly measure specific LV segmental function at multiple time points will allow us to better understand the mechanism and efficacy of a range of left ventriculoplasty procedures that are currently being investigated in clinical trials with other similar device trials on the horizon. The strong correlations of InD improvement with LV volume reductions, along with STE, provide early mechanistic reassurance of the ability for InD to ultimately emerge as the gold-standard for regional LV functional assessment, overcoming many of the shortfalls of TTE. Further clinical validation in both control subjects as well as HFrEF patients pre- and post-left ventriculoplasty along with correlations with clinical and surrogate endpoints of efficacy will be required before InD emerges as the preferred means of screening and monitoring HFrEF patients undergoing assessment for device-based LV interventions.

## Data Availability

The data presented in this study are available on request from the corresponding author. The data are not publicly available due to data-protection.
